# Questioning the “Ease” in disease: Was living with HIV a burden or boost during the first wave of Covid-19 in France? A qualitative study (COVIDHIV)

**DOI:** 10.1371/journal.pone.0295223

**Published:** 2024-03-07

**Authors:** Guillaume Roucoux, Frédérique Thonon, David Zucman, David Rey, Sophie Abgrall, Lars E. Eriksson, Marie Préau, David Michels, Antoine Chéret, Martin Duracinsky

**Affiliations:** 1 ECEVE, UMR-S 1123, Inserm, Université Paris Cité, Paris, France; 2 Patient-Reported Outcomes Research (PROQOL), Unité de Recherche Clinique en Economie de la Santé (URC-ECO), Hôpital Hôtel-Dieu, AP-HP, Paris, France; 3 Foch Hospital, Suresnes, France; 4 Trait d’Union–Strasbourg University Hospitals, Strasbourg, France; 5 Hôpital Antoine Béclère, Service de Médecine Interne, Clamart, France; 6 UVSQ, INSERM U1018, CESP, Université Paris-Saclay, Le Kremlin-Bicêtre, France; 7 Karolinska Institute, Solna, Sueden; 8 Inserm Unit 1296 « Radiations: Defense, Health, Environment », Lyon, France; 9 Lyon 2 Lumière University, Lyon, France; 10 AIDES (French HIV/AIds and Viral Hepatites Organization), Pantin, France; 11 Laboratoire de Recherche Communautaire, Coalition PLUS, Pantin, France; 12 Outpatient Medicine Service, University Hospital, Pointe-à-Pitre, Guadeloupe, France; 13 Internal Medicine Unit, Cochin Hospital, Paris, France; 14 Internal Medicine Unit, Le Kremlin Bicêtre Hospital, Bicêtre, France; Faculty of Medicine, Saint-Joseph University, LEBANON

## Abstract

**Introduction:**

Clinical research has focused on risk factors and treatment for severe acute respiratory syndrome coronavirus 2 (SARS-COV-2), particularly in people with a comorbidity including the human immunodeficiency virus (HIV), but little attention has been paid to the care pathway. This article aims to show how living with HIV may have been a biopsychosocial burden or boost in care pathways for Covid-19.

**Method:**

People living with HIV (PLHIV) from 9 clinical centers were invited to participate in this qualitative study. The sampling was purposive with a maximum variation in their sociodemographic profiles. Semi-structured interviews were conducted until data saturation, then coded for thematic analysis, using an inductive general approach.

**Results:**

We interviewed 34 PLHIV of which 20 had SARS-COV-2 once. They were 24 males, 26 born in France; median age: 55. Twenty had a CD4 number above 500, and all were on antiretroviral therapy (ART). HIV appeared as a burden when Covid-19 symptoms reminded HIV seroconversion, fear of contamination, and triggered questions about ART effectiveness. HIV was not considered relevant when diagnosing Covid-19, caused fear of disclosure when participants sought SARS-COV-2 testing, and its care in hospitals was disrupted by the pandemic. ART-pill fatigue caused avoidance for Covid-19 treatment. As a boost, living with HIV led participants to observe symptoms, to get advice from healthcare professionals, and screening access through them. Some participants could accept the result of screening or a clinical diagnosis out of resilience. Some could consider ART or another drug prescribed by their HIV specialist help them to recover from Covid-19.

**Conclusion:**

Living with HIV could function as a burden and/or a boost in the care pathways for Covid-19, according to patients’ relationship to their HIV history, comorbidities and representation of ART. Covid-19 in PLHIV needs further qualitative study to gain a more comprehensive assessment of the pandemic’s consequences on their lives and coping strategies.

## Introduction

On March 11^th^, 2020, the World Health Organization declared severe acute respiratory syndrome coronavirus 2 (SARS-COV-2), the cause of Covid-19, a pandemic. In several countries, medical research had focused, at the beginning, on populations with a chronic disease, including people who are infected by the human immunodeficiency virus (HIV). On July 13^th^, 2022 France, which has 67 million inhabitants, including 173,000 people living with HIV (PLHIV) [1], had more than 32.5 million SARS-COV-2 cases, 151,000 deaths, and 81% of its population fully vaccinated [2].

Even though systematic reviews and meta-analyses are available [3–9], the epidemiological characteristics, signs, and clinical outcomes of SARS-COV-2 in PLHIV still need to be documented. Most publications identify increased Covid-19 severity in PLHIV compared to the general population or hospitalized patients. Covid-19 symptoms in PLHIV cover a broad spectrum [10], but do not differ from those observed in the general population [11]. Because of their diversity, Covid-19 symptoms could lead general practitioners (GP) to miss diagnosis [12, 13]. Screening access differed according to sex, ethnicity, age, economic status, region, metabolic disorder as well as familiarity with the medical environment [13–20]. Some studies showed a higher risk of hospitalization for PLHIV with SARS-CoV-2 compared to people without HIV [11, 21], mostly correlated with the progression of immunodeficiency and/or the absence of viral suppression [22]. Others have contradicting results [16, 23, 24]. Antiretrovirals (ART) have sparked controversy regarding their potential protection against severe Covid-19 [8–10, 25–28]. Some physicians changed the hospitalized patients’ ART [13, 29–31], and others did not initiate it to prevent an immune reconstitution inflammatory syndrome [32].

Finally, research provided valuable information of Covid-19 in PLHIV’ care pathways. But there is also a need to better understand the patients’ lived experience of the disease, and the pandemic. A qualitative approach is well adapted to research this topic [33]. Though to-date, the study of the subjective experience of SARS-COV-2 symptoms, diagnosis, screening, and treatment in PLHIV in France and worldwide is minimal in the literature. Knowing how PLHIV experienced care pathways for Covid-19 could help situate clinical data into a more comprehensive perspective. While doing so in this article, our aim is to observe how living with HIV may have been a biopsychosocial burden or boost in care pathways for Covid-19.

## Materials and methods

This qualitative study was part of the French COVIDHIV cohort: a multicenter, historico-prospective clinical study, with physiopathological characterization for Covid-19 in PLHIV; with mixed methods [34]. For the pathophysiological study, only PLHIV with proven Covid-19 were considered. For the qualitative study, all patients were considered.

### Population and setting for the qualitative study

Nine clinical centers included participants in the study. Inclusion criteria were: 1) living with HIV; 2) having health insurance scheme, or state medical aid; 3) consent given via signature to collect data for research. Exclusion criteria were: 1) pregnant women; 2) adults under trusteeship; or 3) less than 18 years old.

**Fig 1:**
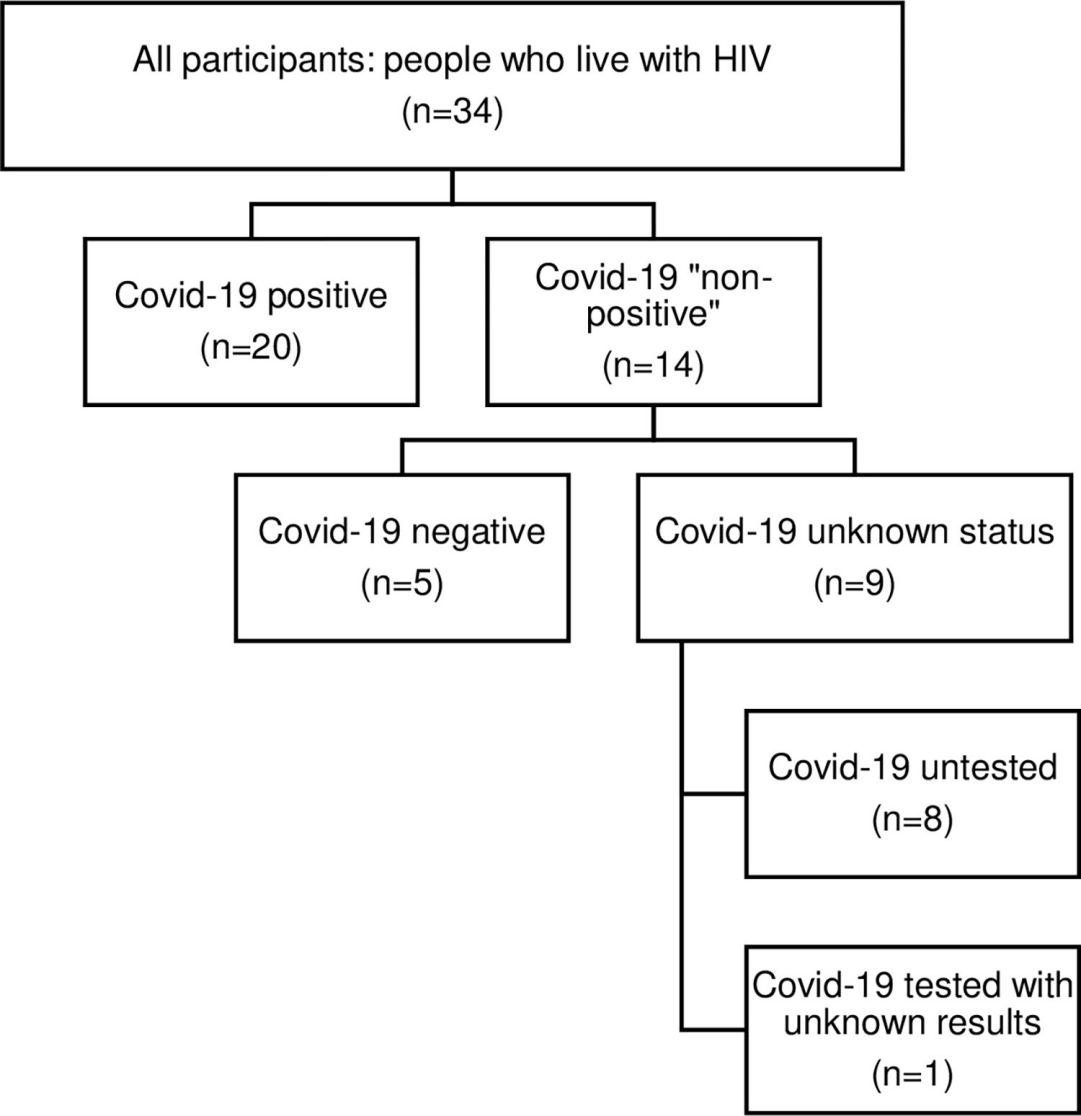
Participants’ inclusion according to their Covid-19 test situation at the time of the interview.

The sampling was purposive with maximum variation [35, 36] in socio-demographic characteristics (age, gender, country of birth, sexual orientation, and professional status). According to the French COVIDHIV cohort inclusion process, participants were divided in two groups according to their Covid-19 status: positive and negative. Recruitment was carried out by a clinical study technician and a researcher. The researcher contacted HIV specialists asking them to invite their patients to participate in the study. The researcher communicated the participant’s anonymity code to the technician. The latter contacted the participant and scheduled the interview. The technician and the researcher contacted each other regularly to monitor the inclusions and the diversification of the sample. Maintaining an anonymous inclusion table made it possible to follow the socio-demographic characteristics of the sample. Women and non-French-born participants were more difficult to include, as it is usual in France due to its HIV sociodemographic barriers. Also, non-screened cases made us redefine the group of Covid-19 negative participants into "non-positive," which included negative (n = 7) and participants with "no result," who had not been tested for Covid-19 (n = 6) or had not received the results (n = 1) at the time of the interview (Fig 1). Since it had no other consequence, our sample remained purposive.

From June to September 2020, during an in-person follow-up or phone consultation, HIV specialists asked patients if they had been sick since January 2020, tested for Covid-19, and wanted to participate in the study. One lead researcher frequently monitored inclusion with a clinical research assistant who facilitated contact between researchers, physicians, and anonymous participants.

### Data collection

One-to-one semi-structured interviewing deemed the most suitable approach to study the lived experience of Covid-19 care pathways. Two interview guides with similar topics were tailored for each group during a brainstorming session, due to the lack of literature and the aim to be more inductive. Since the scarcity of scientific literature at that time, guides were composed of topics by three trained researchers. The guides questioned the perception of vulnerability to Covid-19, information, symptoms, clinical diagnosis, screening, treatment, lockdown, and preventative measures, in open-ended ways. The objective was to elicit participants’ social and psychological experiences for each theme. The interview guides also covered topics to facilitate comparisons between the participants’ experiences of the Covid-19 and HIV infections, respectively, with regard to diagnosis, symptoms, positive status disclosure, fear of transmission, and the perception of death.

Researchers (GR, MD, FT) conducted interviews in French between June 12th and September 10th, 2020 at hospitals (n = 6) and over the phone (n = 28). Participants’ verbal consent was requested once more by each interviewer and recorded at the beginning of the interview. During interviews, they also noted in a time-frame document elements of the patient’s care pathway (symptoms, clinical course, diagnosis and screening dates, treatment duration), medical consultations and significant social events that did not happen due to the pandemic. Interviews were voice-recorded with the consent of the participant. Data collection proceeded until saturation was reached–when additional interviews did not elicit new themes or categories, which was determined by consensus between the three researchers. This was the result of weekly team meetings on the inclusions and content of the interviews, and a meeting dedicated to the codebook.

### Data analysis

Team members transcribed the interviews verbatim and marked audible emotional expressions such as smiling, laughter, sorrow, and doubt. The three interview-researchers independently coded the first five interviews using Word (2019) or Sonal software (v.2.1.41). They then compared results, merged or separated codes when necessary, creating a coding guide. Researchers could not measure inter-coder agreement insofar as the softwares used for coding were different and did not have that option. The main discrepancy observed was in the coding depth of the three different coders. They did not look for discrepancies in the content of the interviews, only in the composition and organization of the codebook. During the meeting dedicated to the codebook, it was decided 1) to keep the major descriptive themes in order to more easily identify the stage of the care pathway in question (symptoms, diagnosis, screening, treatments); 2) to preserve the coding finesse of the lead researcher since he was in charge of the rest of the study). Once he had coded the subsequent interviews, the lead researcher presented the final results to the research team for validation of the themes. This qualitative study adopted a general inductive approach as a framework [37], which led to thematic analysis [38].

### Ethical aspects

The ethical and regulatory review boards of *le Comité de Protection des Personnes Ile de France VIII* required by the recruiting site and the study sponsor have reviewed and approved the research project (IRB ref: 20 04 06; approval 2020-A00984-35). Prior inclusion, participants were given an information and consent note. Their verbal consent was requested by physicians, and audio-recorded by researchers at the beginning of each interview. No identifying data were collected by researchers for any patient.

## Results

### Sociodemographic

Thirty-four individuals participated in the qualitative study. Twenty had at least one positive Covid-19 screening result before the interview, and none were still positive at the time of the interview (positive group), and 14 never had a positive Covid-19 screening result or screening experience at all before the interview (non-positive group). Twenty-four were male–including 18 men who have sex with men–and 10 were female (no participant self-identified as transgender). Their median age was 55. Twenty-six were born in France. Twenty-one lived in the Paris area and 11 in eastern France, which were the two regions that were most impacted by Covid-19 during the first wave. Their HIV diagnosis (HIV Dx) had occurred between 1985 and 2019. All were monitored for HIV and receiving ART (Table 1). Two participants were hospitalized for Covid-19.

**Table 1:** Sociodemographic and clinical characteristics.

	Occurrences	Covid-19 positive participants	Covid-19 non-positive participants
*N =*	34	20	14
**Gender**			
Men	24	15	9
Women	10	5	5
**Age**			
< 40 years old	2	0	2
40–49 years old	11	5	6
50–59 years old	11	10	1
60–69 years old	6	5	1
70–79 years old	4	1	3
**Country of birth**			
France	26	15	11
Another country	8	5	3
*Sub-Saharan Africa*	*5*	*3*	*2*
*South America*	*2*	*1*	*1*
*Middle East*	*1*	*1*	*0*
**Cultural background**			
European	24	15	9
Sub-Saharan African	6	3	3
North African	1	0	1
Middle Eastern	1	1	0
South American	2	1	1
**Region of residence**			
Paris area	21	12	9
East of France	11	8	3
Mostly out of France	2	1	1
**Level of education**			
No education	3	1	2
Elementary school	2	2	0
Middle or high school	12	8	4
College	16	9	7
*Missing data*	*1*	*0*	*1*
**Professional situation**			
With a job	20	13	7
Looking for a job	4	2	2
Neither in labor force, nor retired	2	0	2
Retired	8	5	3
**Marital/family status**			
Single without children	21	12	9
Single with children	1	0	1
In a childless couple	9	6	3
In a couple with children	3	2	1
**Time elapsed since HIV diagnosis**			
< 5 years	4	2	2
5–9 years	4	3	1
10–19 years	14	8	6
20–29 years	6	3	3
30–39 years	6	4	2
**Mode of HIV transmission**			
Homo or bisexual	16	10	6
Heterosexual	14	8	6
Injection-drug use	2	1	1
Other	2	1	1
**Latest CD4 numbers before inclusion**			
< 200	2	1	1
200–499	11	6	5
≥ 500	20	12	8
*Missing data*	*1*	*1*	*0*
**With antiretroviral therapy**	34	20	14
**Symptoms (resembling those) of Covid-19**			
With	22	19	3
Without	12	1	11
**Clinical diagnosis (for Covid-19)**			
By the general practitioner	9	9	0
By the paramedics	4	4	0
By the HIV specialist	4	4	0
By a non-healthcare professional	2	2	0
None	15	1	14
**Covid-19 screening methods**			
Polymerase Chain Reaction	19	15	4
Serology	15	13	2
Chest scan	3	3	0
None	7	Non applicable	7
**Hospitalizations for Covid-19**	2	2	Non applicable

### Data collection

Interviews lasted 64 minutes on average. The interviews of positive participants were longer than the others (71 vs. 49 minutes). Data saturation was observed by the three researchers involved in interviewing and coding triangulation on the 20th interview for the positive group and the 14th for the non-positive one. Timeframes were completed for all. Even though the topic of hospitalization was very detailed in the guide and occurred rarely, researchers did not modify the interview guides, since interviews did not elicit major changes in the expected topics.

### Thematic analysis

**Fig 2:**
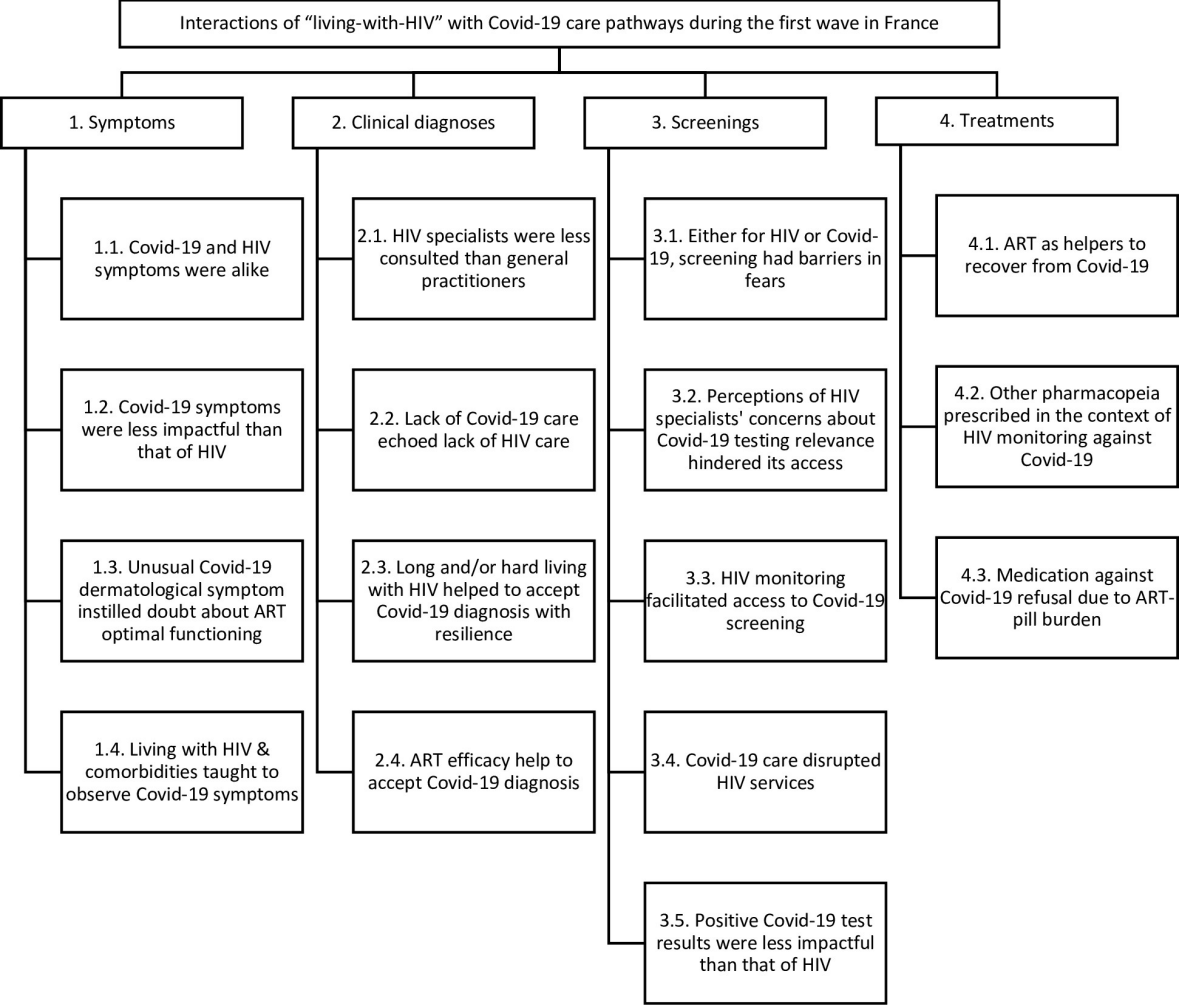
Results of the thematic analysis.

This article focuses on care pathways for Covid-19 (symptoms, clinical diagnoses, screenings, treatments), and the ways living with HIV interacted with them. Seventeen subthemes emerged from the analysis (Fig 2).

#### 1. Symptoms

*1*.*1*. *Covid-19 and HIV symptoms were alike*. A fever was one of the most common symptoms for Covid-19 reported by participants. Three participants also spontaneously spoke of having a fever while describing their HIV seroconversion. One man (≈55 years old [yo]; HIV Dx ≈2010) correlated having a fever as a symptom of the two infections: “after the [HIV] contamination I had a fever for a few days, a bit like Covid. Ultimately, both viruses are alike.” A woman (≈70 yo; HIV Dx ≈1990) highlighted the similarity of the two infections in the symptoms’ manifestation: “we know when we have symptoms [for Covid-19], like for HIV.” A man (age 60; HIV DX ≈1985) noted that individuals could be asymptomatic with both viruses.

*1*.*2*. *Covid-19 were less impactful than that of HIV*. The same woman (≈ 70 yo; HIV Dx ≈1990) also expressed that Covid-19 and HIV symptoms could differ, but in intensity: “[HIV] was catastrophic. So that [Covid-19] was really nothing to me. It was a little flu. HIV was something else. […] Because I was always tired. I did not stop [working], I even got shingles”.

*1*.*3*. *Unusual Covid-19 dermatological symptoms instilled doubt about ART optimal functioning*. Two Covid-19 positive participants reported having had skin issues. A woman (≈45 yo; HIV Dx ≈1995) had doubt about having Covid-19 symptoms. A man (≈55 yo; HIV Dx ≈2015) reported: “I had dry skin on my ankles and calves. After a few days of [topical] non-treatment, the skin scaled as if it had reacted to a sunburn. So, I don’t know if it was the HIV therapy that did this. Or is it the effects of Covid?”

*1*.*4*. *Living with HIV and comorbidities taught to observe Covid-19 symptoms*. Two men (≈60 yo, HIV Dx ≈1990; ≈55 yo, HIV Dx ≈2010) declared having developed the habit to observe symptoms thanks to their medical histories. As the first one put it: “with all my pathologies, I developed a form of observation of all my symptoms. And God knows I have some. And not only HIV.”

#### 2. Clinical diagnoses

*2*.*1*. *HIV specialists were less consulted than general practitioners*. Most participants reported receiving their clinical diagnosis for Covid-19 from their general practitioner (GP) rather than their HIV specialist (HIV SP). One woman (≈70 yo; HIV Dx ≈2010) consulted her GP “*because [she] believed [she had] to consult them when [she had] other [health] problems [than HIV]*.” A man (≈55 yo; HIV Dx ≈2015) thought his HIV SP was less available than the GP due to the saturation of hospital departments to care for Covid-19 cases. Four men (≈55 yo, HIV Dx ≈2010; ≈55 yo, HIV Dx ≈2015; ≈50 yo, HIV Dx ≈2000; ≈45 yo, HIV Dx ≈2005) and one woman (≈60 yo; HIV Dx ≈1995) reported that they received misdiagnosis from the GP, which retrospectively was Covid-19 to them.

*2*.*2*. *Lack of Covid-19 care echoed lack of HIV care*. All participants but two who sought a consultation for Covid-19 symptoms could do so without difficulty, even at the GP’s practice. Two men (both ≈55 yo; HIV Dx ≈2015) reported not being considered as needing proper medical attention from a GP. One expressed the perception of the GP’s fear of becoming infected with SARS-COV-2 without acknowledging the participant’s poor health regarding both HIV and the Covid-19 symptoms. As he narrated:

“He even had a certain attitude in relation to the [Covid-19] symptoms and then, the fact of having no documents, no more explanations [to give me], I felt left alone! […] I had a CD4 count that was [below 200], so really, I had an immune system ‘in the basement’. […] I said to him: ‘Have you seen my file? […] the viral load [sic] is [below 200] so…’ ‘Well, if you have a problem you call [the emergency line]’.”

The other participant encountered difficulties obtaining a medical certificate from the GP to justify sick leave due to Covid-19. Taking the initiative to meet the GP at his practice, described as “empty,” the participant finally obtained the certificate. The participant thought that the GP was afraid of being infected with SARS-COV-2, because of his age and another physician died of Covid-19 in the city. Also, regarding HIV, the participant added about his GP: “He is, I would say, very quiet. I sincerely think that he has a hard time accepting this type of patient.”

*2*.*3*. *Long and/or hard living with HIV helped to accept Covid-19 diagnosis with resilience*. A few participants reflected their life with HIV when approaching Covid-19 diagnosis. One woman (≈70 yo; HIV Dx ≈1990) and one man (≈60 yo, HIV Dx ≈1995) focused on the “shock” of the HIV diagnosis in comparison of Covid-19:

“When you are told that you have HIV, you immediately say to yourself ‘that’s it’, that’s the end. […] It’s obviously a shock […] over time, HIV […] I completely integrated it. […] I no longer have that fear. […] Illness or death tomorrow […] or in fifteen years, it will happen when it will, but it’s not Covid-19 that made me react by saying ’oh dear’, that’s not it at all. So maybe it’s the fact of being infected with HIV for a very long time and going through several phases that made me integrate things.” (man)

One woman (≈40 yo; HIV Dx ≈2000) who was not tested for Covid-19, stated: "[HIV] was so horrible that I could take it [Covid-19]. […] All these phases made me reasonable, […] almost Zen.” One man (≈50 yo; HIV Dx ≈2010), like another man (≈55 yo; HIV Dx ≈2015) who described himself as “a fighter”, expressed his resilience while facing a severe Covid-19 infection:

“My reaction [to Covid-19 diagnosis], was: ‘we have to fight’, that’s all, I was thinking about that. When I was told that it was serious, I said to myself that I still had a lot of things to do and it was not my time: ’Here you go! I survived a deadly disease [HIV], I will not be bothered by a deadly little virus now”.

*2*.*4*. *ART efficacy helped to accept Covid-19 diagnosis*. Two women (≈60 yo; HIV Dx ≈2000; ≈60 yo; HIV Dx ≈1995) and one man (≈55 yo; HIV Dx ≈2015) relied on the ART efficacy in preserving their immune systems to face Covid-19 diagnosis: “I had heard […] that they had experimented [SARS-COV-2 cure] with Kaletra, knowing this therapy, for me, I think it helped me [to face Covid-19]” (second woman).

#### 3. Screenings

*3*.*1*. *Either for HIV or Covid-19*, *fears were barriers for screening*. One man (≈55 yo; HIV Dx ≈2010) expressed to not “understand why people are reluctant to go for the [Covid-19] test, but it is, for HIV, the same thing.” One woman (≈70 yo; HIV Dx ≈1990) evoked the fear of the syringe as a means to transmit viruses. Several participants were tested within their HIV department. The COVIDHIV study was an opportunity for a healthcare professional who had the possibility of being tested at work to keep her HIV status confidential. Another woman (≈40 yo; HIV Dx ≈2015) was afraid that her HIV status could be revealed by SARS-COV-2 serology.

*3*.*2*. *Perceptions of HIV specialists’ concerns about Covid-19 testing relevance hindered its access*. Most GPs systematically referred to screening. But, according to two men (≈45 yo, HIV Dx ≈2005; ≈70 yo; HIV Dx ≈2010), HIV SP did not consider screening relevant due to the absence of symptoms or a “concern.” The test reliability did not convince the HIV SP of another man (≈60 yo; HIV Dx ≈1990) at the very beginning of the pandemic. One man (≈50 yo; HIV Dx ≈2000) thought the specialist was considering the high costs of mass screening.

*3*.*3*. *HIV monitoring facilitated access to Covid-19 screening*. The COVIDHIV study was also an opportunity for some participants to have their first screening test. Blood samples for monitoring HIV facilitated the Covid-19 serology: “I came: usual blood test, plus a little bit for Covid” (man; ≈55 yo; HIV Dx ≈2010). “Blood sampling,” a short version of “blood samplings for HIV,” was often used to name Covid-19 serology. Participants with this screening scheme received their results for both viruses as they usually get for HIV. Also, “we pay nothing here, and they gave us everything,” added one woman (≈70 yo; HIV Dx ≈1990).

*3*.*4*. *Covid-19 care disrupted HIV services*. One man (≈60 yo; HIV Dx ≈2005) regretted the contradictory management of concomitant Covid-19 testing and HIV care. The HIV department contacted him to cancel the routine appointment three times while inviting him to get tested for Covid-19. He had to negotiate with the medical staff to get both simultaneously.

*3*.*5*. *Positive Covid-19 test results were less impactful than that of HIV*. Covid-19 results did *“*nothing” to the majority, or was a *“*confirmation,*”* a “surprise,” or a “relief.” One man (≈60 yo; HIV Dx ≈1990) self-named a “virus bank,” explained his non-negative reaction as being “used to healthcare.” Another one (≈65 yo; HIV Dx ≈2005) said: “It [screening result] didn’t do much to me since I’m already HIV positive.” All participants reported a hard time processing their HIV screening results though. One man (≈60 yo, HIV Dx ≈2010) who was waiting for his results at the time of the interview explained that, in comparison, the Covid-19 one “is neutral information: I have it, I got it. Well, it’s not like HIV, indeed if there is no treatment, well there is a treatment [for HIV], now here I am on my feet, life goes on.”

*3*.*6*. *Positive Covid-19 test results aroused the perception of vulnerability and fear of contaminating*. Two men (in their 40s, HIV Dx ≈2005) reported “anguish” or “panic” when diagnosed with Covid-19 due to a general lack of knowledge of how the virus functions and is transmitted. The fear of transmission echoed the one of HIV for one of them: “I was afraid of contaminating others [with SARS-COV-2], in fact. I was afraid for my mother, honestly […] deeply”. Screened Covid-19 positive months after recovery, one man in his mid-50s, diagnosed with HIV in the 2010s, expressed: “it [the serology result] reminded me once again of my HIV, that I am vulnerable to all viruses.”

#### 4. Treatments

*4*.*1*. *ART as helpers to recover from Covid-19*. The majority of participants had heard of trials using antiretroviral against Covid-19. Their reactions varied between enthusiasm, curiosity, and skepticism. One woman, in her late 50s, diagnosed with HIV in the mid-1990s, reported: “I had heard that they had experimented with [an ART], knowing this therapy, I think it helped me.”

*4*.*2*. *Other pharmacopeia prescribed in the context of HIV monitoring against Covid-19*. A few participants mentioned being reminded by their HIV SP to get vaccinated against the seasonal flu. Two men (≈55 yo, HIV Dx ≈2015; ≈60 yo, HIV Dx ≈2010) wondered if the flu vaccine could have helped them recover from Covid-19 or prevent the development of a severe form of the disease. One man (≈60 yo; HIV Dx ≈1990) mentioned the diphtheria, tetanus and poliomyelitis vaccine in the same way. Two men (both ≈55 yo; HIV Dx ≈2015), one vaccinated against the flu and the other one non-vaccinated, questioned the possibility that vitamin D, prescribed by their HIV SP, could also have had a benefit against SARS-COV-2.

*4*.*3*. *Medication against Covid-19 refusal due to ART-pill burden*. Two SARS-COV-2 positive participants (≈60 yo, HIV Dx ≈1990; ≈60 yo, HIV Dx ≈1985) said having had “no treatment” for Covid-19 but consecutively reported having used ones, as if these were at first not worth noticing. The second one complained: “for Covid, I had no treatment. I had nothing, except advice to follow, [while] HIV treatments were heavy at the beginning, even now, sometimes I’m tired of drugs.”

## Discussion

HIV may have been a burden in the care pathways for Covid-19. Covid-19 symptoms reminded participants of HIV seroconversion (1.1), or confused people with ART malfunctioning (1.3). Some GPs did not consider it relevant when diagnosing Covid-19 (2.2), and HIV follow-up was disrupted by the pandemic (3.4). HIV caused a fear of disclosure when searching to be tested for Covid-19 (3.1), and Covid-19 revived fear of HIV contaminating (3.6). ART-pill fatigue caused Covid-19 non-treatment (4.3). On the contrary, HIV may have been a boost in the care pathways for Covid-19. Some PLHIV have learned to observe symptoms (1.2, 1.4). They could get the advice of two doctors (2.1, 3.2, 4.2), and screening access through them (3.3). Some could accept the result of screening or a clinical diagnosis out of resilience (2.3, 2.4, 3.5), and considered ART or another drug prescribed by the HIV SP help recover from Covid-19 (4.1, 4.2).

To our knowledge, the experience of Covid-19 symptoms as reminders of HIV seroconversion is little documented in the literature. However, Rhodes et al. also interviewed a participant whose Covid-19 test result was consistent with his HIV diagnosis [39]. This finding echoed HIV-related post-traumatic stress disorder from which some PLHIV may suffer, and which the symptoms of another pathology can exacerbate [40]. Symptoms and diagnoses can trigger bad memories with HIV, such as the seroconversion itself, guilt, or fear of HIV transmission. This is something to be careful about when diagnosing a comorbidity in PLHIV. HIV in PLHIV should always be considered as a biological and psychological marker, by healthcare professionals including HIV SP and GPs. During the first wave of the pandemic in France, GPs also felt stressed [41, 42], and the situation obliged them to adapt their activity to include more prevention [43]. The non-considering-HIV attitude of the two GPs reported by our participants is reminiscent of GPs’ attitude towards PLHIV in the 1990s, when only one in ten GPs agreed to care for a PLHIV (without symptoms and a high CD4 count) as a unique provider [44]. It also reminds HIV-related stigmatization in healthcare settings may exist in countries with a high level of HIV knowledge and medical care, and has consequences on quality of care [45]. Moreover, like in many countries [46], HIV services have been disrupted in France. Only one participant experienced frustration on being included in the cohort without being given the opportunity to have his routine HIV visit. Most of the participants’ HIV consultations have been postponed to a calmer phase or in-between two waves, or occurred online, with no ART discontinued deliveries. If the confusion of a Covid-19 symptom with ART malfunctioning did not appear in the literature, ART-pill burden resulting in a diminution of medication is documented, but for long-term medication [47]. One participant expressed ART-pill burden may also diminish short-term medication as well and medication in general. Finally, our results coincide with those of Nitpolprasert et al. about the fear expressed by PLHIV that Covid-19 screenings would disclose HIV status, and contribute to stigmatization [48]. Even if the national contexts differ, it seems important to remind PLHIV in follow-up consultation that the Covid-19 screening methods cannot show this result, and professionals trained in screening who have access to PLHIV health information to keep it confidential. Also, PLHIV with Covid-19 feared more HIV stigmatization than Covid-19 stigmatization [49].

Living with HIV has helped some participants to care for themselves, being more attentive to any potential signs of health issues, which could be considered as a self-management strategy for coping with adverse events [50]. Also, according to Brandão et al. to have support from health professionals is one of the coping strategies used by PLHIV [51]. In France, most PLHIV are monitored by a GP for any health issues while HIV is monitored by a specialist. As shown by Linnemayr et al, connections to medical services have enabled PLHIV to be better prepared to avoid complications from Covid-19 [52], and it helped our participants to access to screening.

The development of Covid-19 screenings was both crucial in the first wave of the pandemic and the source of implementation difficulties [53]. Access to Covid-19 screening tests, at an early stage of the pandemic via the cohort study, could motivate participation in PLHIV, for personal curiosity and scientific progress. Participating in HIV research can provide PLHIV with personal and social benefits [54]. Contrary to the results of Hall et al. [55], only one participant expressed fear for her life when she learned of her positive Covid-19 result (due to her age). However, as in Hall et al.’s study, most HIV diagnoses were more impactful than that of Covid-19, due in particular to the presence of the first diagnosis potentially facilitating the possibility of being cured of the second. Moreover, as in our study, Hall et al. also encountered the opposite case, with the similar reason of the uncertainty surrounding Covid-19 as a novel disease versus the significant knowledge of HIV for those being diagnosed with HIV today. Similar to the experiences expressed by some of the participants in the present study, Gwadz et al. and Quinn et al. found that PLHIV who experienced the lethal risk of living with HIV developed resilience to the uncertainty surrounding Covid-19 [20, 56]. Also, consistent with our findings, Hall et al., building on Del Amo et al. [26], reported that knowledge of pharmacological trials of ART against Covid-19 created the feeling of protection in PLHIV. Nonetheless, our results showed that other medication prescribed by the HIV SP played that role as well.

As limitations, only two participants were hospitalized, and only one had a severe form of Covid-19. All participants had well-controlled HIV. Our criterion did not permit inclusion of participants who did not benefit from or were not entitled to health insurance or State medical aid, who are the most precarious; disability or impairment due to age. Focusing primarily on the Parisian and eastern areas, we did not interview persons living in regions less severely affected by Covid-19 during the first wave of the pandemic. Different courses may have taken place where PLHIV are more isolated than our participants, or healthcare facilities are rarer, causing difficulty in diagnosing, screening, and/or treating. We did not interview people whose HIV is solely monitored by a GP or who have no monitoring at all. The absence of common working tools and the decisions taken may constitute methodological limitations of the study. Finally, the study set at an early phase of the pandemic was an opportunity to qualitatively assess its most critical peak, but it did not allow to incorporate its evolutions (i.e. the vaccine), which would contribute to different experiences as well. The pandemic, knowledge, and treatments are still evolving and we learn from other contexts.

This is the first qualitative study in France focusing on PLHIV confronted with the Covid-19 pandemic. Similar to the conclusions made by Hall et al., the results remind us that living with HIV cannot be reduced to the fact of a “virus in a body.” Our results enhance the notion that living with HIV is a biopsychosocial experience that is part of the duration and the relationship with the medical environment. As such, the supposed vulnerability of PLHIV to SARS-COV-2 can not only be restricted to biology. Furthermore, the resources of PLHIV to cope with the infection cannot be solely pharmacological or medico-technical. Further research is needed to understand how living with HIV shapes patient’s agency. Also, the HIV-Covid-19 co-infection simultaneous diagnosis and care remains a line of future research.

## Conclusion

Living with HIV could, from a biopsychosocial perspective, function both or either as a burden and a boost in the care pathways for Covid-19, according to patients’ relationship to their HIV history and comorbidities, ART, GP and HIV specialist. Health professionals are significant contributors to this in the way they care for PLHIV. Covid-19 in PLHIV needs further qualitative research to gain a more comprehensive evaluation of the pandemic’s consequences on their lives, its impacts, and their coping strategies.

### The COVIDHIV scientific and executive committee

Pr Cécile Goujard, Pr Laurence Meyer, Dr Martin Duracinsky, Mrs Fatoumata Waggeh, Pr Lamiae Grimaldi, Dr Véronique Avettand Fenoël, Dr Delphine Dujardin, Dr Anne Marie Roque Afonso, Pr Christine Rouzioux, Mrs Marianne L’Henaff, Dr Evguenia Krastinova, Dr Claudine Duvivier, Dr Gilles Pialoux, Pr Karine Lacombe, Mrs Rémonie Seng, Pr Marie Préau, Pr James W. Griffith, Pr Lars E Erisksson, Mr David Michels, Mrs Albertine Pabingui.

## Supporting information

S1 Checklist COREQ guideline checklist. (DOCX)
