# Supplementary material for: Questioning the “Ease” in disease: Was living with HIV a burden or boost during the first wave of Covid-19 in France? A qualitative study (COVIDHIV)
Source: PLoS One. 2024 Mar 7;19(3):e0295223. doi: 10.1371/journal.pone.0295223 (PMC10919596; doi:10.1371/journal.pone.0295223)
Supplement: S1 Checklist — (DOCX) [file pone.0295223.s001.docx]

COREQ Guideline checklist:

| **Domain 1: Research team and reflexivity** | **Personal characteristics** | |
| --- | --- | --- |
|  | 1. Which author(s) conducted the interview? | FT conducted 1 interview  GR conducted 31 interviews  MD conducted 2 interviews |
|  | 1. What were the researcher’s credentials? | FT: PhD in public health  GR: PhD candidate in sociology  MD: MD-PhD |
|  | 1. What was their occupation at the time of the study? | FT: employed as researchers at INSERM UMR1123  GR: employed as researchers at INSERM UMR1123  MD: team leader of PROQOL INSERM UMR1123 |
|  | 1. Was the researcher male or female? | FT: female, GR: male, MD: male, |
|  | 1. What experience or training did the researcher have? | FT: training in qualitative methods for public health  GR: training in qualitative methods for ethnology & sociology  MD: training in qualitative methods for public health |
|  | **Relationship with participants** | |
|  | 1. Was a relationship established prior to study commencement | No relationship was established priori to study commencement |
|  | 1. What did the participants know about the researcher? | The participants only knew that the researchers were public health and social sciences researchers |
|  | 1. What characteristics were reported about the interviewer/facilitator? | See 7 |
| **Domain 2: Study design** | **Theoretical framework** | |
|  | 1. What methodological orientation was stated to underpin the study? | General Inductive Approach, Weberian Interpretative Sociology & Thematic Analysis |
|  | **Participant selection** | |
|  | 1. How were the participants selected? | Participants were selected by their HIV doctor who agreed to participate to the COVIDHIV cohort study (See “Methods, Population and settings” subsection) |
|  | 1. How were the participants approached? | Doctors proposed to patients to participate in the study face to face during HIV consultation at hospital, or over the phone (in case of teleconsultation) |
|  | 1. How many participants were in the study? | 34 |
|  | 1. How many participants refused to participate or dropped out? Why? | 4. One dropped out because she did not want to be voice-recorded, one because of the theorical one hour length of the interview, and two were no more reachable over the phone. |
|  | **Setting** | |
|  | 1. Where was the data collected? | 28 interviews were carried out over the phone with audio recording, and 6 interviews were carried out face to face (in hospitals, right after the routine HIV consultation) |
|  | 1. Was anyone else present besides the participants and researcher? | No one else was present during interviews |
|  | 1. What are the important characteristics of the sample? | Characteristics of participants are reported in detail in “Results, sociodemography” |
|  | **Data collection** | |
|  | 1. Were questions, prompts, guides provided by the author? Was it pilot tested? | 2 interview guides prepared before starting the interview. There was no adaptation during the interview phase |
|  | 1. Were repeat interviews carried out? Details | No repeat interviews |
|  | 1. Did the researcher use audio or visual recording to collect the data? | Audio-recording of all interviews |
|  | 1. Were field notes made during and/or after the interview or focus group? | Fieldnotes taken during all interviews |
|  | 1. What was the duration of interviews or focus groups? | Interviews lasted between 12 minutes to 2 hours 7 minutes (average: 1h03, median: 1h01). |
|  | 1. Was data saturation discussed? | FT, GR and MD discussed data saturation |
|  | 1. Were transcripts returned to participants for comments and/or correction? | Transcripts were not returned to participants |
| **Domain 3: Analysis and findings** | **Data analysis** | |
|  | 1. How many data coders coded the data? | 3. FT, GR and MD independently coded 5 interviews for data triangulation. GR coded all other interviews |
|  | 1. Did authors provide a description of the coding tree? | Partially. No coding tree *per see* is provided, but a thematic diagram displays the major themes resulting from the analysis and details the four themes that are central in the article |
|  | 1. Were themes identified in advance or derived from the data? | 7 themes were anticipated through the interview guide, but the final coding tree derived from data |
|  | 1. What software, if applicable, was used to manage the data? | Sonal (2.1.41) |
|  | 1. Did participants provide feedback on the findings? | Yes, 2, who were interested in knowing the study results, accepted to review the article before submission |
|  | **Reporting** | |
|  | 1. Were participant quotations presented to illustrate the themes/findings? Was each quotation identified? | Participants’ quotations are presented to illustrate findings. They are identified in the article and in appendix 3: Table of quotations |
|  | 1. Was there consistency between the data presented and the findings? | Yes |
|  | 1. Were major themes clearly presented in the findings? | Yes |
|  | 1. Is there a description of diverse cases or discussion of minor themes? | Yes |
